# Efficacy and Safety of Dexmedetomidine Premedication in Balanced Anesthesia: A Systematic Review and Meta-Analysis in Dogs

**DOI:** 10.3390/ani11113254

**Published:** 2021-11-14

**Authors:** Shi-Yue Pan, Gang Liu, Jia-Hao Lin, Yi-Peng Jin

**Affiliations:** Department of Clinical Veterinary Medicine, College of Veterinary Medicine, China Agricultural University, 2 Yuanmingyuan West Road, Haidian District, Beijing 100193, China; shiyue.pan@foxmail.com (S.-Y.P.); liugang_0402@126.com (G.L.); jiahao_lin@cau.edu.cn (J.-H.L.)

**Keywords:** dexmedetomidine, sedation and pain score, hemodynamic effects, dog, meta-analysis

## Abstract

**Simple Summary:**

Dexmedetomidine, on account of its potent sedative and analgesic properties, is commonly used in balanced anesthesia of small animal anesthesia; however, concerns regarding its cardiovascular effects prevent its full adoption into veterinary clinical practice. We conducted this meta-analysis to determine the effects of dexmedetomidine on sedation, analgesia, cardiovascular and adverse reactions in dogs compared to other premedications. The outcomes included sedation score, pain score, heart rate, systolic arterial blood pressure, mean arterial blood pressure and the incidence of adverse effects. Thirteen studies were included in this meta-analysis. The results showed that dexmedetomidine provides a satisfactory sedative and analgesic effect in balanced anesthesia of dogs. After dexmedetomidine premedication, dogs experienced lower heart rate and higher blood pressure within an acceptable range. The combinations in balanced anesthesia and routes of delivering drugs would affect heart rate, systolic arterial blood pressure, and mean arterial blood pressure of dogs. Before using dexmedetomidine, an animal’s cardiovascular status should be fully considered.

**Abstract:**

Dexmedetomidine is commonly used in small animal anesthesia for its potent sedative and analgesic properties; however, concerns regarding its cardiovascular effects prevent its full adoption into veterinary clinical practice. This meta-analysis was to determine the effects of dexmedetomidine on sedation, analgesia, cardiovascular and adverse reactions in dogs compared to other premedications. Following the study protocol based on the Cochrane Review Methods, thirteen studies were included in this meta-analysis ultimately, involving a total of 576 dogs. Dexmedetomidine administration probably improved in sedation and analgesia in comparison to acepromazine, ketamine and lidocaine (MD: 1.96, 95% CI: [−0.08, 4.00], *p* = 0.06; MD: −0.95, 95% CI: [−1.52, −0.37] *p* = 0.001; respectively). Hemodynamic outcomes showed that dogs probably experienced lower heart rate and higher systolic arterial blood pressure and mean arterial blood pressure with dexmedetomidine at 30 min after premedication (MD: −13.25, 95% CI: [−19.67, −6.81], *p* < 0.0001; MD: 7.78, 95% CI: [1.83, 13.74], *p* = 0.01; MD: 8.32, 95% CI: [3.95, 12.70], *p* = 0.0002; respectively). The incidence of adverse effects was comparable between dexmedetomidine and other premedications (RR = 0.86, 95% CI [0.58, 1.29], *p* = 0.47). In summary, dexmedetomidine provides satisfactory sedative and analgesic effects, and its safety is proved despite its significant hemodynamic effects as part of balanced anesthesia of dogs.

## 1. Introduction

Dexmedetomidine, a highly selective α-2 receptor agonist with potent sedative and analgesic properties, is commonly used as premedication in balanced anesthesia in small animal clinical medicine [1,2]. It has been reported to provide sedative properties paralleling natural sleep, with minimal respiratory depression in rats [3]. In addition, it has a significant impact on anesthetic requirements, such as a sparing effect on the minimal alveolar concentration (MAC) of inhaled anesthetic [4]. Combined with opioid analgesics, dexmedetomidine can effectively reduce the dosage of the combinations [5]. In recent years, there is increasing evidence supporting its synergetic effects, alternative routes of administration and organ-protective effects against ischemic and hypoxic injury [6,7].

Despite its widespread clinical use and research in human beings, concerns on the cardiovascular effects of dexmedetomidine prevent its full adoption into veterinary practice [8]. This might be owing to a greater sensitivity of dogs for the vasoconstrictor effect of α-2 receptor agonists compared to humans [9]. In dogs, α-2 receptor agonists may induce systolic impairment due to their peripheral vascular action [10]. Animals may even experience bradycardia and transient hypertension in the early stages after dexmedetomidine premedication [11].

Therefore, the purpose of this meta-analysis was to examine the efficacy (sedation and analgesia) and safety (cardiovascular and adverse reactions) of dexmedetomidine compared to other premedications as a part of balanced anesthesia in dogs.

## 2. Materials and Methods

### 2.1. Literature Search Strategy

This meta-analysis was based on the Preferred Reporting Items for Systematic Reviews and Meta-Analyses (PRISMA) and the Cochrane Review Methods [12]. We searched electronic databases PubMed and CAB Abstracts up to March 2021, and the following search terms were applied: (dog OR “dogs” [Mesh] OR canine *) AND (“dexmedetomidine” [Mesh] OR MPV-1440 OR MPV 1440 OR MPV1440 OR precedex) AND (sedation OR pain OR “analgesia” [Mesh] OR “anesthesia” [Mesh] OR balanced anesthesia OR cardiovascular OR “hemodynamics” [Mesh] OR circulatory OR heart rate OR blood pressure) AND (safety OR safe OR adverse effect * OR effect * OR undesirable effect * OR tolerability OR toxicity OR reaction * OR disease *). A filter of clinical trials was applied to the results. No language restrictions were placed on the search. Finally, the references of all articles retrieved from the search were manually reviewed and Google Scholar was queried for any relevant trials not already identified using the strategy described above.

### 2.2. Outcomes

Trials comparing dexmedetomidine to sedative or analgesic in premedication, investigating sedation and pain outcomes in balanced anesthesia of dogs were included in the present meta-analysis. Extracted outcomes were selected according to the standard approach described in some meta-analysis of dexmedetomidine premedication in humans [13,14,15,16]. The primary outcomes were sedation score and pain score after premedication. The sedation score was performed using a composite simple descriptive score after dexmedetomidine administration. Full consciousness and alertness were scored as 0 and unconsciousness as 20. If the score scales were different in some studies, the data were converted according to the scoring standard used by Grint and others [17]. The pain score was performed at 120 min after the dexmedetomidine premedication, when the operation was nearly ended. Perioperative pain score was evaluated according to the short form of Glasgow composite pain score (GCPS) [18]. The maximum pain score was achieved with 24 points. Secondary outcomes were hemodynamic changes, including heart rate (HR), systolic arterial blood pressure (SAP) and mean arterial blood pressure (MAP) at time points of 30 and 60 min after premedication. At this time, the animal was generally under operation in a stable state. We were also interested in the adverse effects, including the incidence of arrhythmia, apnea and rescue analgesia. Extracted trial characteristics included pre-medication of each group, the number of dogs, doses and the route of drug delivery, the medications used to induce or maintain anesthesia and other administration.

### 2.3. Quality Assessment

#### 2.3.1. Assessment of Risk of Bias

We used the Cochrane Collaboration’s Risk of Bias Tool (ROB 2) for randomized controlled trials to assess the methodological quality of these randomized trials [19]. Two authors (S.-Y.P. and G.L.) independently scored the bias, which considers the methods of random sequence generation, allocation concealment, blinding of participants and outcome assessment, incomplete reporting of outcome data, selective reporting and other bias risks, such as special study design. Disagreements were resolved through discussion with a third author (J.-H.L.).

#### 2.3.2. Certainty of Evidence

The Grade of Recommendation, Assessment, Development and Evaluation (GRADE) Working Group system was used to assess the certainty of evidence for each outcome [20].

### 2.4. Data Extraction and Analysis

The study protocol was determined before data extraction and archived in the College of Veterinary Medicine, China Agricultural University. We set premedication with dexmedetomidine as the dexmedetomidine group no matter what the dose or route of administration used. Meanwhile, premedication with other drugs was considered as the comparisons, no matter which drug was used. Following the Cochrane Collaboration Risk of Bias tool, we assessed the included studies.

The outcome variables were the incidence or mean differences between groups. In some studies, the numerical data were extracted from graphs by “WebPlotDigitizer” (online source) [21]. According to the method of Shi J. and Luo D. et al. [22,23], we converted the median, quartile and range into mean and standard deviation before analyzing. All statistical analyses were conducted using the Review Manager software (RevMan version 5.4). The heterogeneity was evaluated by the coefficient I^2^ [24]. If the I^2^ statistic had a value of more than 50%, which presents moderate or high heterogeneity, the random-effects model was used. Otherwise, the fixed-effects model was applied [25].

A subgroup analysis was utilized according to the time points after premedication in an attempt to evaluate how the effect changed over time. In addition, a subgroup analysis was conducted according to the classification of the comparator. The effects caused by routes of administration and the combination of induction agent were also considered. Funnel plots were used to evaluate the risk of publication bias for the outcomes of the studies included. A sensitivity analysis was conducted to assess whether the studies caused high heterogeneity could affect the results. The results were presented as mean difference (MD) for continuous data or risk ratio (RR) for binary variables with 95% confidence interval (CI). A two-sided value of *p* < 0.05 was considered significant.

## 3. Results

### 3.1. Characteristics of Studies

**Figure 1:**
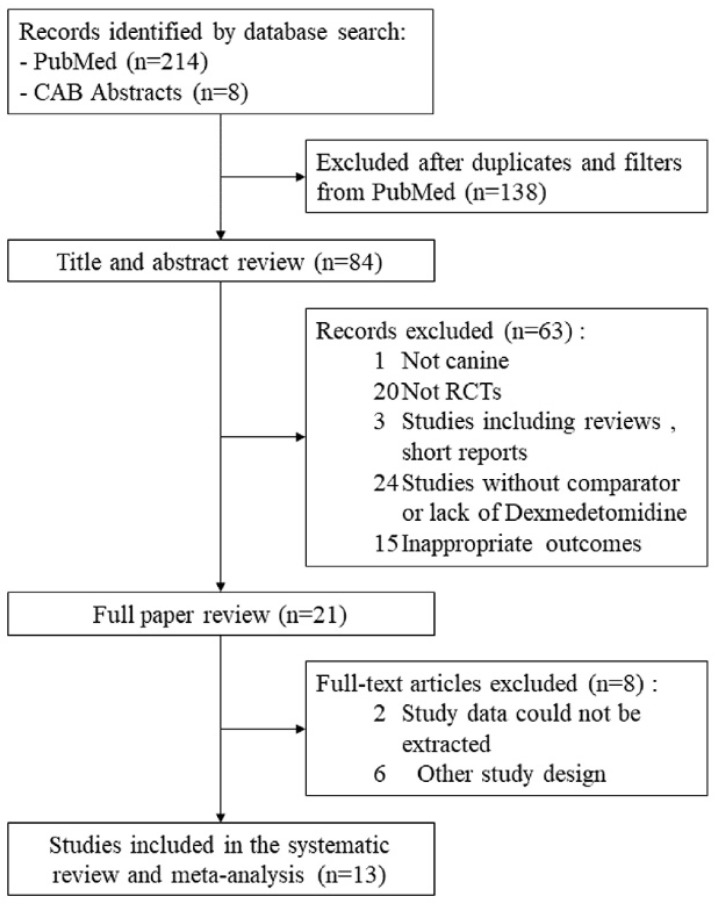
Flow diagram showing literature search results.

Among the 222 trials initially identified from the search strategy, 13 studies were included in this meta-analysis [17,26,27,28,29,30,31,32,33,34,35,36,37], involving a total of 576 dogs of various breeds. Most of the selected dogs were classified as having ASA 1 and ASA 2 physical status. A flow chart to demonstrate the study selection and exclusion process is shown in Figure 1. The included studies were undertaken from 2009–2018.

The authors investigated doses of dexmedetomidine ranging from 1 to 10 μg.kg^−1^ (1 μg.kg^−1^≈25 μg.m^−2^) [17,27,29,31,35], combined with methadone [24,28], ketamine [32,37] or buprenorphine [26,32,35,36] as premedication. Four included trials used alfaxalone to induce or maintain anesthesia [26,29,33,35], and eight used propofol [17,28,30,31,33,34,35,36]. Other interventions than dexmedetomidine were the comparisons. Four studies set acepromazine as the comparison [26,33,35,36], four studies used α-2 receptor agonists as the comparison [28,30,32,37] and five studies had opioids in the comparison [17,27,29,31,34]. Six studies set more than one comparison [30,31,33,34,35,37]. Three articles compared the effects of different doses of dexmedetomidine to the comparisons during balanced anesthesia [17,29,36]. Therefore, the results needed to be analyzed and discussed separately. The characteristics of the included studies are reported in Table 1.

**Figure 2:**
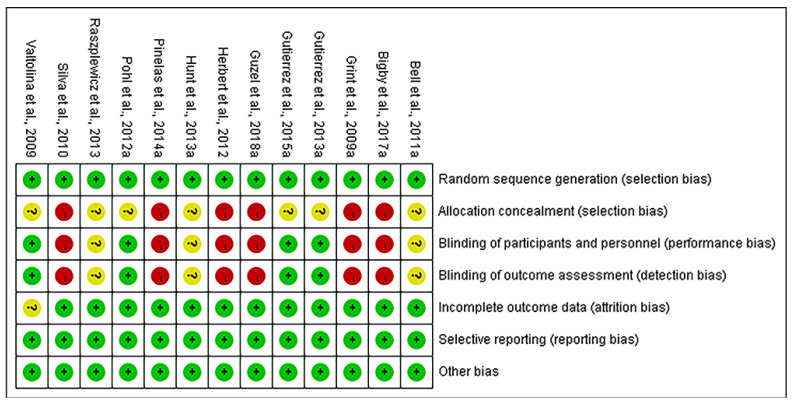
Cochrane Collaboration risk of bias summary. Green circle, low risk of bias; yellow circle, unclear risk of bias; red circle, high risk of bias.

The risk of bias, according to the Cochrane Collaboration Risk of Bias tool, is presented in Figure 2. Some of the studies did not entirely blind participants/personnel/outcome assessment, which were assessed as high risk [17,26,29,32,33,37]. Those who mentioned blinding but did not describe the measures used were rated as unclear risk [28,35,36]. The studies that did not mention the allocation concealment were rated as high risk [17,26,29,32,33,37]. Seven studies mentioned random sequence generation and allocation concealment without further description were rated as unclear risk [27,28,30,31,32,33,34,35,36]. One study disclosed losses to follow-up without analyses were assessed as unclear risk of incomplete outcome data [27].

### 3.2. Primary Outcomes

#### 3.2.1. Sedation Score

**Figure 3:**
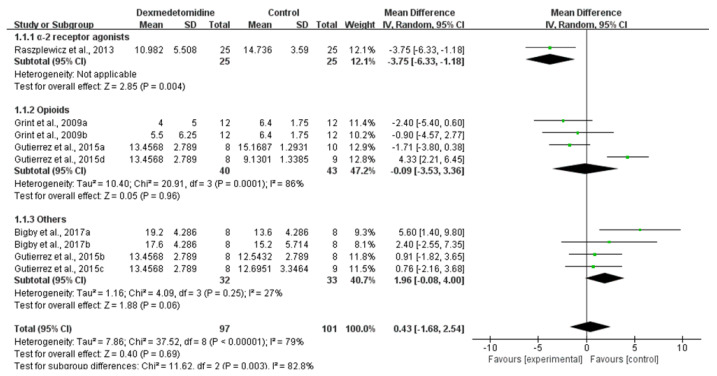
Forest plot of sedation scores between dexmedetomidine and pethidine [16], medetomidine [27], acepromazine [32] and fentanyl, ketamine, lidocaine and butorphanol [33] in balanced anesthesia.

The sedation score was reported in four studies [17,28,33,34]. Dexmedetomidine administration may improve sedation in comparison to other premedications (Low CoE; MD: 0.043; 95% CI: [−1.68, 2.54]; I^2^: 79%; *p* = 0.69, Figure 3). A subgroup analysis of comparisons found that dexmedetomidine administration may result in a slightly reduction in the sedation score in comparison to opioids (MD: −0.09; 95% CI: [−3.53, 3.36]; I^2^: 86%; *p* = 0.96; number of studies 16, 33) and an increase in comparison to acepromazine, ketamine and lidocaine (MD: 1.96; 95% CI: [−0.08, 4.00]; I^2^: 27%; *p* = 0.06; number of studies 33, 34).

#### 3.2.2. Pain Score

**Figure 4:**
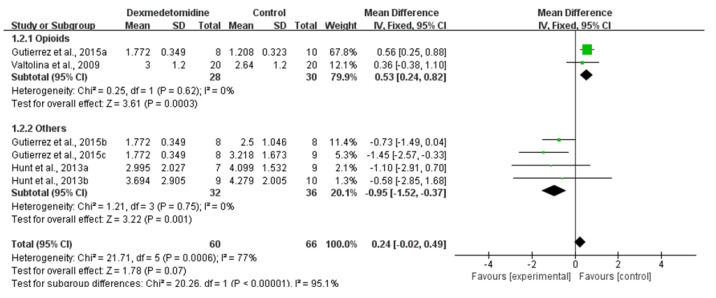
Forest plot of pain scores at 30 min and 120 min after premedication between dexmedetomidine and morphine [27], fentanyl, ketamine and lidocaine [34] and acepromazine [35] in balanced anesthesia.

The pain assessment was reported in three studies [26,33,34]. Dexmedetomidine administration probably improved in analgesia in comparison to other premedications (Moderate CoE; MD: 0.24; 95% CI: [−0.02, 0.49]; I^2^: 77%; *p* = 0.07, Figure 4). A subgroup analysis of comparisons found that analgesia of dexmedetomidine administration is lower in comparison to opioids (MD: 0.53; 95% CI: [0.24, 0.82]; I^2^: 0%; *p* = 0.0003; number of studies 27, 34) and is higher in comparison to acepromazine, ketamine and lidocaine (MD: −0.95; 95% CI: [−1.52, −0.37]; I^2^: 0%; *p* = 0.001; number of studies 34, 35) according to the pain score.

### 3.3. Secondary Outcomes

Eight studies reported the hemodynamic indicators [17,26,27,29,30,31,32,37]. All hemodynamic outcomes were influenced by dexmedetomidine. Due to the high heterogeneity, a subgroup analysis of HR, SAP and MAP at a time point of 30 min after premedication was conducted according to the classification of the comparator. The “α-2 receptor agonists” group included studies used medetomidine, xylazine and detomidine as comparator. The “Opioids” group included studies used morphine, methadone, fentanyl, butorphanol and pethidine as comparator. The “Others” group included studies used acepromazine, ketamine and lidocaine as comparator. A sensitivity analysis was conducted to address the heterogeneity.

#### 3.3.1. HR

**Figure 5:**
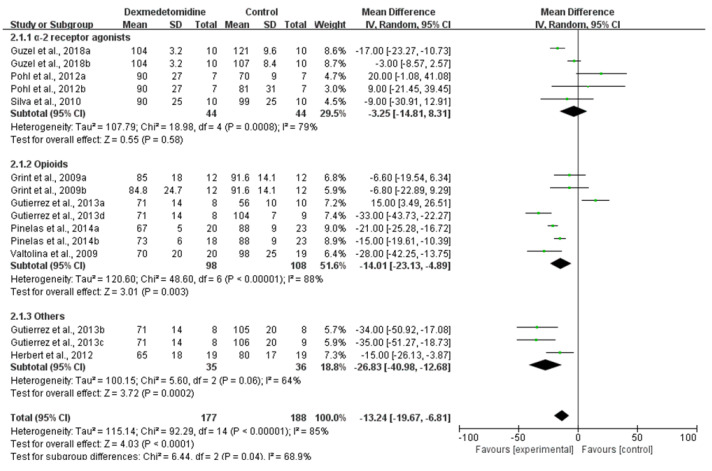
Forest plot of HR at 30 min after premedication between dexmedetomidine and pethidine [17], acepromazine [26], morphine [27], methadone [29], medetomidine, detomidine and xylazine [30,32,37] and fentanyl, ketamine, lidocaine and butorphanol [31] in balanced anesthesia.

The HR of dogs for which dexmedetomidine was used was significantly lower than that of other comparisons (Low CoE; MD: −13.25; 95% CI: [−19.67, −6.81]; I^2^: 85%; *p* < 0.0001; number of studies 17, 26, 27, 29–32, 37; n = 8; Figure 5). A subgroup analysis of comparisons found that the HR of dexmedetomidine administration may results in a slightly reduction in comparison to α-2 receptor agonists (MD: −3.25; 95% CI: [−14.81, 8.31]; I^2^: 79%; *p* = 0.58; number of studies 30, 32, 37) and a significantly reduction in comparison to opioids (MD: −14.01; 95% CI: [−23.13, −4.89]; I^2^: 88%; *p* = 0.003 number of studies 17, 27, 29, 31).

#### 3.3.2. SAP

**Figure 6:**
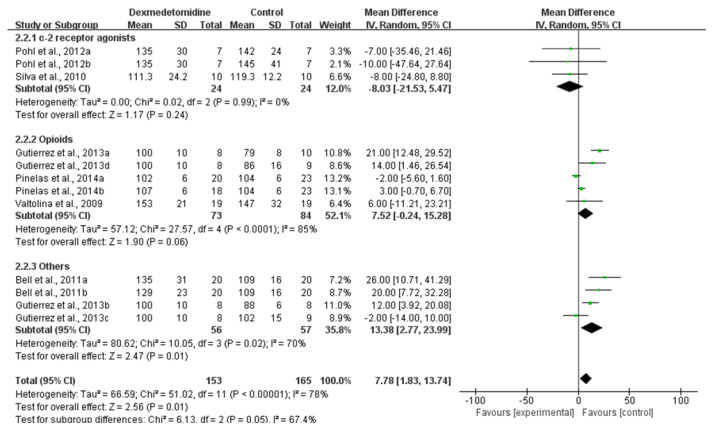
Forest plot of SAP at 30 min after premedication between dexmedetomidine and morphine [27], methadone [29], detomidine and xylazine [30], fentanyl, ketamine, lidocaine and butorphanol [31], medetomidine [32] and acepromazine [36] in balanced anesthesia.

The SAP of dogs for which dexmedetomidine was used was significantly higher than that of other comparisons (Moderate CoE; MD: 7.78; 95% CI: [1.83, 13.74]; I^2^: 78%; *p* = 0.01; number of studies 27, 29–32, 36; n = 6; Figure 6). A subgroup analysis of comparisons found that the SAP of dexmedetomidine administration probably results in a slightly reduction in comparison to α-2 receptor agonists (MD: −8.03; 95% CI: [−21.53, 5.47]; I^2^: 0%; *p* = 0.24; number of studies 29, 31) and a significantly increase in comparison to opioids (MD: 7.52; 95% CI: [−0.24, 15.28]; I^2^: 85%; *p* = 0.06 number of studies 27, 29, 31).

#### 3.3.3. MAP

**Figure 7:**
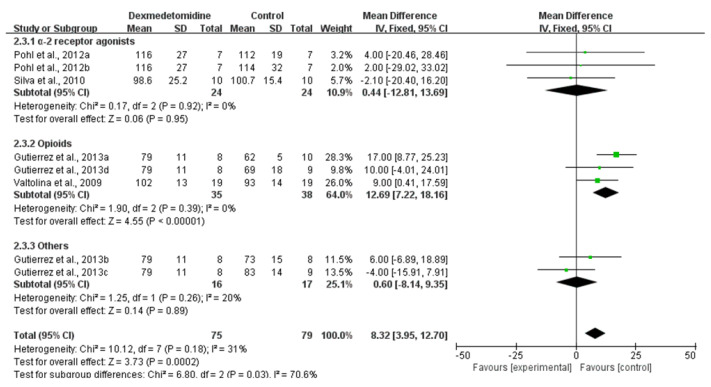
Forest plot of MAP at 30 min after premedication between dexmedetomidine and morphine [27], detomidine and xylazine [30], fentanyl, ketamine, lidocaine and butorphanol [31] and medetomidine [32] in balanced anesthesia.

The MAP of dogs for which dexmedetomidine was used was significantly higher than that of other comparisons (High CoE; MD: 8.32; 95% CI: [3.95, 12.70]; I^2^: 31%; *p* = 0.0002; number of studies 27, 30–32; n = 4; Figure 7). A subgroup analysis of comparisons found that the MAP of dexmedetomidine administration results in a slightly increase in comparison to α-2 receptor agonists (MD: −0.44; 95% CI: [−12.81, 13.69]; I^2^: 0%; *p* = 0.95; number of studies 30, 32) and a significantly increase in comparison to opioids (MD: 12.69; 95% CI: [7.22, 18.16]; I^2^: 0%; *p* < 0.00001 number of studies 27, 31).

The results of HR, SAP and MAP at a time point of 60 min after premedication are shown in Supplementary Figure S1–S6.

### 3.4. Safety Outcome

**Figure 8:**
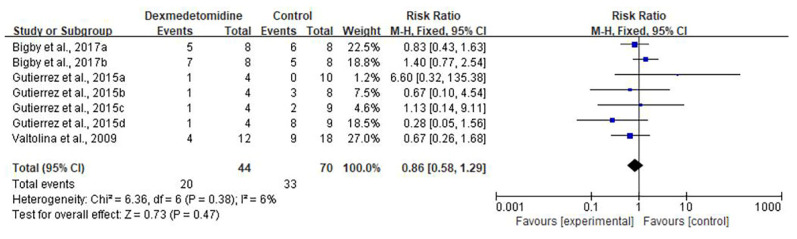
Forest plot of the adverse events between dexmedetomidine and morphine [27], acepromazine [33] and fentanyl, ketamine, lidocaine and butorphanol [34] in balanced anesthesia.

Three studies were included in this session [27,33,34]. There was no difference between dexmedetomidine, morphine, acepromazine, fentanyl, ketamine, lidocaine and butorphanol in regard of adverse events such as apnea, arrhythmias and requirement of rescue analgesia (Moderate CoE; RR = 0.86; 95% CI [0.58, 1.29]; I^2^: 6%; *p* = 0.47; Figure 8). A sensitivity analysis of each outcome was performed. The results showed that none of the studies strongly influenced the outcomes.

**Figure 9:**
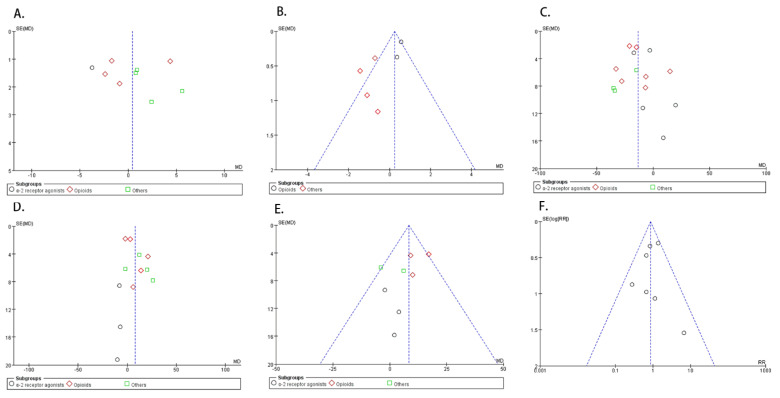
Funnel plot of each outcome between dexmedetomidine and comparisons in balanced anesthesia. (**A**) Sedation scores; (**B**) pain scores; (**C**) HR at 30 min after premedication; (**D**) SAP at 30 min after premedication; (**E**) MAP at 30 min after premedication; (**F**) adverse effects.

The funnel plot of each outcome is shown in Figure 9.

The summary of findings is presented in Table 2.

## 4. Discussion

Based on the result of the meta-analysis of 13 randomized controlled trials (RCTs) with 576 dogs of various breeds, the sedative effect of dexmedetomidine was better than that of acepromazine, ketamine, lidocaine and butorphanol, but inferior to that of pethidine, fentanyl and medetomidine in balanced anesthesia of dogs. Its analgesic effect was better than acepromazine, ketamine and lidocaine but not to the level of opioids. Notably, the comparisons above on the sedative and analgesic effects could be influenced by the dosage, the type and dosage of the combinations, the route of administration and the type of surgery. In small animal clinical medicine, dexmedetomidine is commonly used because it can significantly decrease the MAC of inhaled anesthetics. To increase analgesia, dexmedetomidine can be used in conjunction with opioids. Lower doses of morphine combined with dexmedetomidine may provide analgesia equivalent to or better than a higher dose of morphine alone [38].

After subgroup analysis of HR, SAP and MAP according to the classification of the comparator, the heterogeneity within some subgroups was still high. This could be owing to the inconsistent results of the effects of other drugs compared to dexmedetomidine. For example, fentanyl had a stronger effect on lowering HR and SAP of dogs than dexmedetomidine, while dexmedetomidine can decrease the HR and SAP of dogs better than methadone. Even if the same drug was used, the results were significantly different due to the difference in dosage and route of administration, which contributed to high heterogeneity. More research that meets the criteria is warranted. According to the sensitivity analysis, the results of HR and SAP would not change when studies were excluded that increased heterogeneity.

Intriguingly, although the animals in the dexmedetomidine group experienced low HR 30 and 60 min after premedication, the studies assessed suggested that the HR of dogs did not decrease significantly combined with propofol. Propofol is a short-acting intravenous anesthetic that can be used to produce sedation, as well as to induce and maintain anesthesia. The literature showed that HR of dogs after propofol induction was significantly higher, while the MAP was significantly lower [39]. It was reported that blood pressure can remained within an acceptable range in dogs given dexmedetomidine and anesthetized with propofol [8]. This could account for the drug–drug interaction that dexmedetomidine might inhibit the metabolism of propofol and improve the cardiovascular indicators of animals. Moreover, sedation with dexmedetomidine and induction with propofol can prolong the period of anesthesia and reduce the amount of all components in balanced anesthesia.

Additionally, the routes of administration influenced the effect of dexmedetomidine on hemodynamics. The blood pressure increased caused by dexmedetomidine was due to the activation of α-1 and α-2 adrenergic receptors in the vascular endothelium to produce extensive vasoconstriction [4]. In terms of pharmacokinetics, the absorption and distribution of the drug after extravascular administration are not as fast as intravenous injection [40]. Theoretically, intramuscular or other routes of administration can slow down the diffusion of the drug to the vascular endothelium, which can reduce the cardiovascular effects. A recent study suggested that oral transmucosal administration of dexmedetomidine and methadone combination provided a satisfactory level of sedation with less pronounced cardiorespiratory effects, which could be considered as a useful option for those dogs whose cardiovascular stability should be preserved [41].

As for the safety, dogs experienced lower HR and higher SAP and MAP with dexmedetomidine at 30 min after premedication; however, none needed treatment for bradycardia and hypertension [42]. Using low doses of atipamezole was an approach for treating dexmedetomidine-induced bradycardia in general anesthesia, which may also reduce arterial blood pressure via α-2 adrenoceptor blockade [43,44]. For the most part, dexmedetomidine is safe and effective for small animals with ASA 1 and ASA 2 physical status, as well as some irritable animals and even wildlife.

There were several limitations to this meta-analysis. Firstly, this meta-analysis used only HR and blood pressure as cardiovascular indicators, while it would be more comprehensive to include right atrial pressure, mean pulmonary artery pressure, cardiac index, stroke volume index, stroke vascular resistance index and other parameters [45]. Secondly, it is difficult to conduct a more detailed subgroup analysis, because there are many drugs used in balanced anesthesia, which would affect the validity of the results. In addition, some included trials had many groups with a small sample size, which decreased the statistical power within these studies [46]. Thirdly, the heterogeneity of this study was high, even if a subgroup analysis was conducted. It may be related to the dosage, type and dosage of the combinations, route of administration and type of surgery in the studies. Finally, the insufficient data related to adverse events demonstrated the need of more RCTs. Although the included studies were all RCTs, some of them did not entirely blind participants/personnel/outcome assessment due to safety concerns, increasing the risk of performance and detection bias. Therefore, well-controlled randomized studies are warranted.

## 5. Conclusions

In conclusion, this meta-analysis found that dexmedetomidine provides a satisfactory sedative and analgesic effect in balanced anesthesia of dogs. After dexmedetomidine premedication, dogs experienced lower heart rate and higher blood pressure within an acceptable range. No difference was detected between dexmedetomidine and other premedications regarding adverse events such as apnea, arrhythmias and the requirement of rescue analgesia.

## Figures and Tables

**Table 1:** Characteristics of the included studies.

Study	Year ^1^	Country	Premedication	Dose ^2^	Route	Group	n	Surgical Procedure	Induce/Maintain Anesthesia	Other Administration	Funding	Conflict of Interest
Herbert et al.	2012	UK	dexmedetomidine	≈10 μg·kg^−1^	IM	DEX	19	OH	alfaxalone	buprenorphine	unknow	unknow
			acepromazine	0.05 mg·kg^−1^		CON	19					
Valtolina et al.	2009	NL	dexmedetomidine	≈1 μg·kg^−1^	IV	DEX	20	exploratory laparotomy, thoracotomy or orthopedic surgery	isoflurane	sufentanil	Orion Pharma Animal Health	unknow
			morphine	≈0.1 mg·kg^−1^		CON	20					
Pinelas et al.	2014a	UK	dexmedetomidine	1 μg·kg^−1^	IM	DEX	20	diagnostic, orthopedic or elective soft tissue surgical procedures	alfaxalone	methadone	unknow	unknow
	2014b		dexmedetomidine	3 μg·kg^−1^		DEX	18					
			methadone	0.2 mg·kg^−1^		CON	23					
Pohl et al.	2012	BRA	dexmedetomidine	2 μg·kg^−1^	EPI	DEX	7	OH	propofol	lidocaineand adrenalineand acepromazine	unknow	unknow
	2012a		xylazine	0.25 mg·kg^−1^		CON	7		and isoflurane			
	2012b		detomidine	30 μg·kg^−1^		CON	7					
Gutierrez et al.	2013	MEX	dexmedetomidine	1 μg·kg^−1^	IV	DEX	8	OH	propofol	unknown	unknow	sponsored by a scholarship provided by PROMEP-SEP
	2013a		fentanil	5 μg·kg^−1^		CON	10		and isoflurane			
	2013b		Ketamine	1 mg·kg^−1^		CON	8					
	2013c		lidocaine	2 mg·kg^−1^		CON	9					
	2013d		butorphanol	0.4 mg·kg^−1^		CON	9					
Silva et al.	2010	BRA	dexmedetomidine	20 μg·kg^−1^·h^−1^	CRI	DEX	10	OH	Midazolam and ketamine	levomepromazine and buprenorphine	unknow	unknow
			medetomidine	30 μg·kg^−1^·h^−1^		CON	10					
Bigby et al.	2017a	AU	dexmedetomidine	5 μg·kg^−1^	IM	DEX	8	elective neutering procedures	alfaxalone	methadone	unknow	no conflict of interest
			acepromazine	0.05 mg·kg^−1^		CON	8					
	2017b		dexmedetomidine	5 μg·kg^−1^	IM	DEX	8		propofol	methadone		
			acepromazine	0.05 mg·kg^−1^		CON	8					
Gutierrez et al.	2015	MEX	dexmedetomidine	1 μg·kg^−1^	IV	DEX	8	OH	propofol	unknown/none	Ministry of Public Education of Mexico PROMEP-SEP	unknow
	2015a		fentanil	5 μg·kg^−1^		CON	10		and isoflurane			
	2015b		ketamine	1 mg·kg^−1^		CON	8					
	2015c		lidocaine	2 mg·kg^−1^		CON	9					
	2015d		butorphanol	0.4 mg·kg^−1^		CON	9					
Bell et al.	2011a	UK	dexmedetomidine	≈2.5 μg·kg^−1^	IM	DEX	20	elective procedures	propofol	buprenorphine	unknow	unknow
	2011b		dexmedetomidine	≈5 μg·kg^−1^		DEX	20					
			acepromazine	30 μg·kg^−1^		CON	20					
Guzel et al.	2018	TUR	dexmedetomidine	3 μg·kg^−1^	IV	DEX	10	surgical procedures due to miscellaneous conditions	none	ketamine	unknow	unknow
	2018a		medetomidine	10 μg·kg^−1^		CON	10					
	2018b		xylazine	0.5 mg·kg^−1^		CON	10					
Raszplewicz et al.	2013	UK	dexmedetomidine	0.005 mg·kg^−1^	IM	DEX	25	elective diagnostic imaging procedures	propofol	butorphanol	Janssen Animal Health	unknow
			medetomidine	0.01 mg·kg^−1^		CON	25					
Grint et al.	2009a	UK	dexmedetomidine	5 μg·kg^−1^	IM	DEX	12	routine OH or castration	propofol	none	Orion Pharma	unknow
	2009b		dexmedetomidine	10 μg·kg^−1^		DEX	12		and isoflurane			
			pethidine	5 mg·kg^−1^		CON	12					
Hunt et al.	2013a	UK	dexmedetomidine	≈10 μg·kg^−1^	IM	DEX	7	elective surgeries	propofol	buprenorphine	Orion Pharma	unknow
			acepromazine	0.03 mg·kg^−1^		CON	9					
	2013b		dexmedetomidine	≈10 μg·kg^−1^	IM	DEX	9		alfaxalone	buprenorphine		
			acepromazine	0.03 mg·kg^−1^		CON	10					

^1^ Using letters after ‘Year’ to distinguish more than one group in the Review Manager software. ^2^ In some studies, 25 ug.m^−2^ ≈ 1 μg.kg^−1^; 25 mg.m^−2^ ≈ 1 mg.kg^−1^. IM, intramuscular injection; IV, intravenous injection; EPI, epidural injection; CRI, constant rate intravenous infusion; CON, control; DEX, dexmedetomidine; OH, ovariohysterectomy.

**Table 2:** Summary of findings.

Certainty Assessment							No. of Patients		Effect		Certainty
Findings	No. of Studies	Risk of Bias	Inconsistency	Indirectness	Imprecision	Other Considerations	Dexmedetomidine	Comparisons	Relative (95% CI)	Absolute (95% CI)	
Sedation score	4 RCTs	serious ^1^	serious ^2^	not serious	not serious	none	97	101	-	MD 1.06 higher (1.44 higher to 3.57 higher)	⨁⨁◯◯ LOW
Pain score–120 min after premedication	3 RCTs	not serious	serious ^2^	not serious	not serious	none	60	66	-	MD 0.34 higher (1.09 higher to 0.41 higher)	⨁⨁⨁◯ MODERATE
HR–30 min after premedication	8 RCTs	serious ^1^	serious ^2^	not serious	not serious	none	177	188	-	MD 13.24 lower (19.67 lower to 6.81 lower)	⨁⨁◯◯ LOW
HR–60 min after premedication	6 RCTs	serious ^1^	serious ^2^	not serious	not serious	none	133	144	-	MD 16.86 lower (26.47 lower to 7.24 lower)	⨁⨁◯◯ LOW
SAP–30 min after premedication	6 RCTs	not serious	serious ^2^	not serious	not serious	none	153	165	-	MD 7.78 higher (1.83 higher to 13.74 higher)	⨁⨁⨁◯ MODERATE
SAP–60 min after premedication	5 RCTs	not serious	serious ^2^	not serious	not serious	none	113	124	-	MD 3.59 higher (3.68 lower to 10.87 higher)	⨁⨁⨁◯ MODERATE
MAP–30 min after premedication	4 RCTs	not serious	not serious	not serious	not serious	none	75	79	-	MD 7.27 higher (1.61 higher to 12.93 higher)	⨁⨁⨁⨁ HIGH
MAP–60 min after premedication	4 RCTs	not serious	serious ^2^	not serious	not serious	none	75	78	-	MD 8.06 higher (1.25 higher to 14.87 higher)	⨁⨁⨁◯ MODERATE
Adverse effects	3 RCTs	not serious	not serious	serious ^3^	not serious	none	20/44 (45.5%)	33/70 (47.1%)	RR 0.86 (0.58 to 1.29)	66 fewer per 1000 (from 198 fewer to 137 more)	⨁⨁⨁◯ MODERATE

^1^ The majority of the studies had high risks in allocation concealment and blinding. ^2^ Coefficient I^2^ above 50%. ^3^ The adverse effect only reflected by the incidence of arrhythmia, apnea and rescue analgesia. CI, Confidence interval; MD, Mean difference; RR, Risk ratio.

## Data Availability

The data presented in this study can be made available on direct request to the corresponding author.

## Supplementary Materials

The following are available online at https://www.mdpi.com/article/10.3390/ani11113254/s1, Figure S1: Forest plot of HR at 60 min after premedication between dexmedetomidine and control group in balanced anesthesia, Figure S2 Forest plot of SAP at 60 min after premedication between dexmedetomidine and control group in balanced anesthesia, Figure S3: Forest plot of MAP at 60 min after premedication between dexmedetomidine and control group in balanced anesthesia, Figure S4: Funnel plot of HR at 60 min after premedication between dexmedetomidine and control group in balanced anesthesia, Figure S5: Funnel plot of SAP at 60 min after premedication between dexmedetomidine and control group in balanced anesthesia. Figure S6: Funnel plot of MAP at 60 min after premedication between dexmedetomidine and control group in balanced anesthesia.
