# Supplementary material for: Efficacy and Safety of Dexmedetomidine Premedication in Balanced Anesthesia: A Systematic Review and Meta-Analysis in Dogs
Source: Animals (Basel). 2021 Nov 14;11(11):3254. doi: 10.3390/ani11113254 (PMC8614454; doi:10.3390/ani11113254)
Supplement: Supplementary file 1 [file animals-11-03254-s001.zip › animals-1455469-supplementary.pdf]

Supplementary Material

1 Supplementary Figures

Study or Subgroup	Dexmedetomidine Mean	Dexmedetomidine SD	Dexmedetomidine Total	Control Mean	Control SD	Control Total	Weight	Mean Difference IV, Random, 95% CI
**2.19.1 α-2 receptor agonists**								
Pohl et al., 2012a	82	14	7	76	29	7	7.0%	6.00 [-17.86, 29.86]
Pohl et al., 2012b	82	14	7	73	26	7	7.5%	9.00 [-12.88, 30.88]
Silva et al., 2010	93	31	10	99	25	10	6.8%	-6.00 [-30.68, 18.68]
**Subtotal (95% CI)**			**24**			**24**	**21.2%**	**3.55 [-9.94, 17.05]**
Heterogeneity: Tau² = 0.00; Chi² = 0.85, df = 2 (P = 0.65); I² = 0%								
Test for overall effect: Z = 0.52 (P = 0.61)								
**2.19.2 Opioids**								
Gutierrez et al., 2013a	86	19	8	61	21	10	8.4%	25.00 [6.49, 43.51]
Gutierrez et al., 2013d	86	19	8	115	7	9	9.7%	-29.00 [-42.94, -15.06]
Pinelas et al., 2014a	70	10	20	98	10	23	11.6%	-28.00 [-33.99, -22.01]
Pinelas et al., 2014b	73	8	18	98	10	23	11.7%	-25.00 [-30.51, -19.49]
Valtolina et al., 2009	67	24	20	96	30	19	8.8%	-29.00 [-46.11, -11.89]
**Subtotal (95% CI)**			**74**			**84**	**50.2%**	**-19.23 [-31.16, -7.30]**
Heterogeneity: Tau² = 144.49; Chi² = 29.47, df = 4 (P < 0.00001); I² = 86%								
Test for overall effect: Z = 3.16 (P = 0.002)								
**2.19.3 Others**								
Gutierrez et al., 2013b	86	19	8	109	17	8	8.6%	-23.00 [-40.67, -5.33]
Gutierrez et al., 2013c	86	19	8	99	12	9	9.3%	-13.00 [-28.32, 2.32]
Herbert et al., 2012	80	16	19	126	17	19	10.6%	-46.00 [-56.50, -35.50]
**Subtotal (95% CI)**			**35**			**36**	**28.6%**	**-28.00 [-49.60, -6.40]**
Heterogeneity: Tau² = 308.62; Chi² = 13.67, df = 2 (P = 0.001); I² = 85%								
Test for overall effect: Z = 2.54 (P = 0.01)								
**Total (95% CI)**			**133**			**144**	**100.0%**	**-16.86 [-26.47, -7.24]**
Heterogeneity: Tau² = 197.49; Chi² = 65.22, df = 10 (P < 0.00001); I² = 85%								
Test for overall effect: Z = 3.44 (P = 0.0006)								
Test for subgroup differences: Chi² = 8.62, df = 2 (P = 0.01), I² = 76.8%								

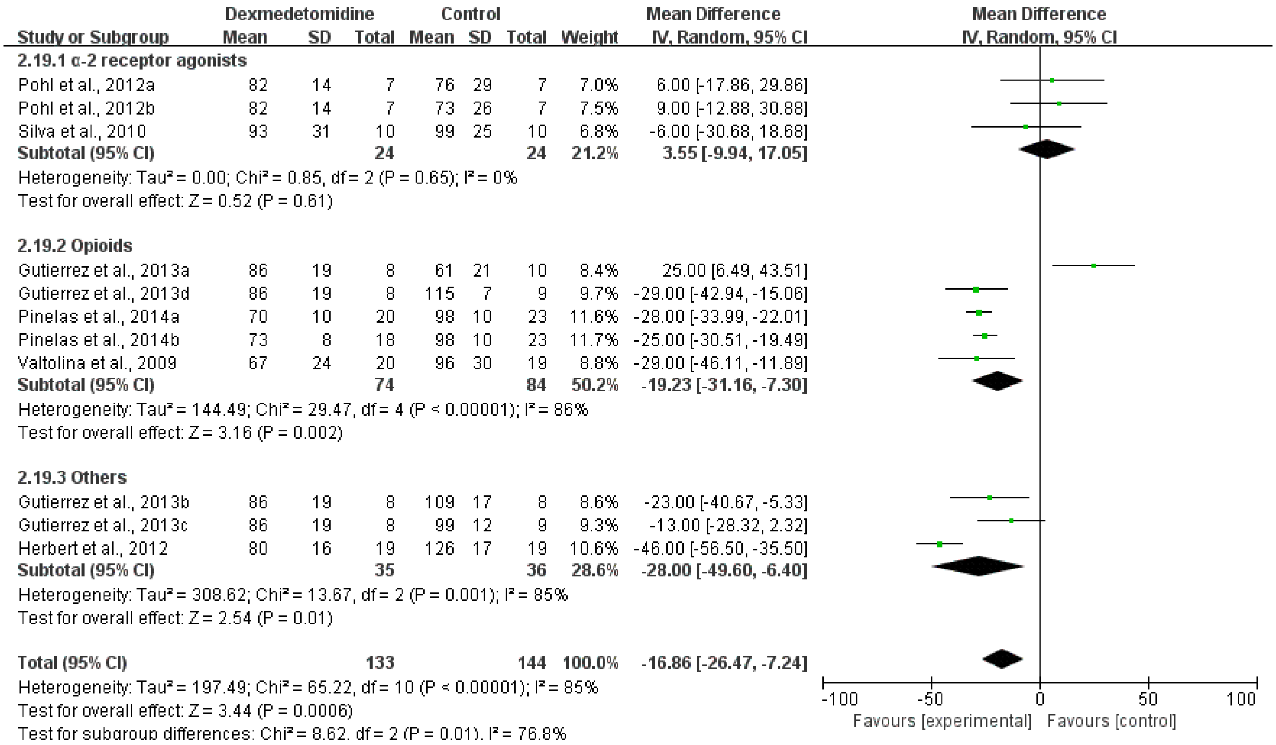

Figure S1. Forest plot of HR at 60 min after premedication between dexmedetomidine and control group in balanced anesthesia.

Study or Subgroup	Dexmedetomidine Mean	SD	Total	Control Mean	SD	Total	Weight	Mean Difference IV, Random, 95% CI
**2.20.1 α-2 receptor agonists**								
Pohl et al., 2012a	111	20	7	129	34	7	4.4%	-18.00 [-47.22, 11.22]
Pohl et al., 2012b	111	20	7	152	44	7	3.3%	-41.00 [-76.80, -5.20]
Silva et al., 2010	125.3	20.1	10	120.1	16.8	10	8.7%	5.20 [-11.04, 21.44]
**Subtotal (95% CI)**			**24**			**24**	**16.4%**	**-14.27 [-41.04, 12.50]**
Heterogeneity: Tau² = 372.79; Chi² = 6.15, df = 2 (P = 0.05); I² = 67%								
Test for overall effect: Z = 1.04 (P = 0.30)								
**2.20.2 Opioids**								
Gutierrez et al., 2013a	131	9	8	113	11	10	12.4%	18.00 [8.76, 27.24]
Gutierrez et al., 2013d	131	9	8	115	22	9	9.0%	16.00 [0.33, 31.67]
Pinelas et al., 2014a	101	8	20	107	6	23	14.7%	-6.00 [-10.28, -1.72]
Pinelas et al., 2014b	106	8	18	107	6	23	14.6%	-1.00 [-5.44, 3.44]
Valtolina et al., 2009	149	22	19	151	32	18	8.0%	-2.00 [-19.79, 15.79]
**Subtotal (95% CI)**			**73**			**83**	**58.6%**	**4.07 [-4.71, 12.85]**
Heterogeneity: Tau² = 73.16; Chi² = 26.02, df = 4 (P < 0.0001); I² = 85%								
Test for overall effect: Z = 0.91 (P = 0.36)								
**2.20.3 Others**								
Gutierrez et al., 2013b	131	9	8	125	11	8	12.0%	6.00 [-3.85, 15.85]
Gutierrez et al., 2013c	131	9	8	115	8	9	12.9%	16.00 [7.86, 24.14]
**Subtotal (95% CI)**			**16**			**17**	**25.0%**	**11.40 [1.63, 21.17]**
Heterogeneity: Tau² = 28.76; Chi² = 2.35, df = 1 (P = 0.12); I² = 58%								
Test for overall effect: Z = 2.29 (P = 0.02)								
**Total (95% CI)**			**113**			**124**	**100.0%**	**3.59 [-3.68, 10.87]**
Heterogeneity: Tau² = 89.20; Chi² = 48.91, df = 9 (P < 0.00001); I² = 82%								
Test for overall effect: Z = 0.97 (P = 0.33)								
Test for subgroup differences: Chi² = 3.56, df = 2 (P = 0.17), I² = 43.8%								

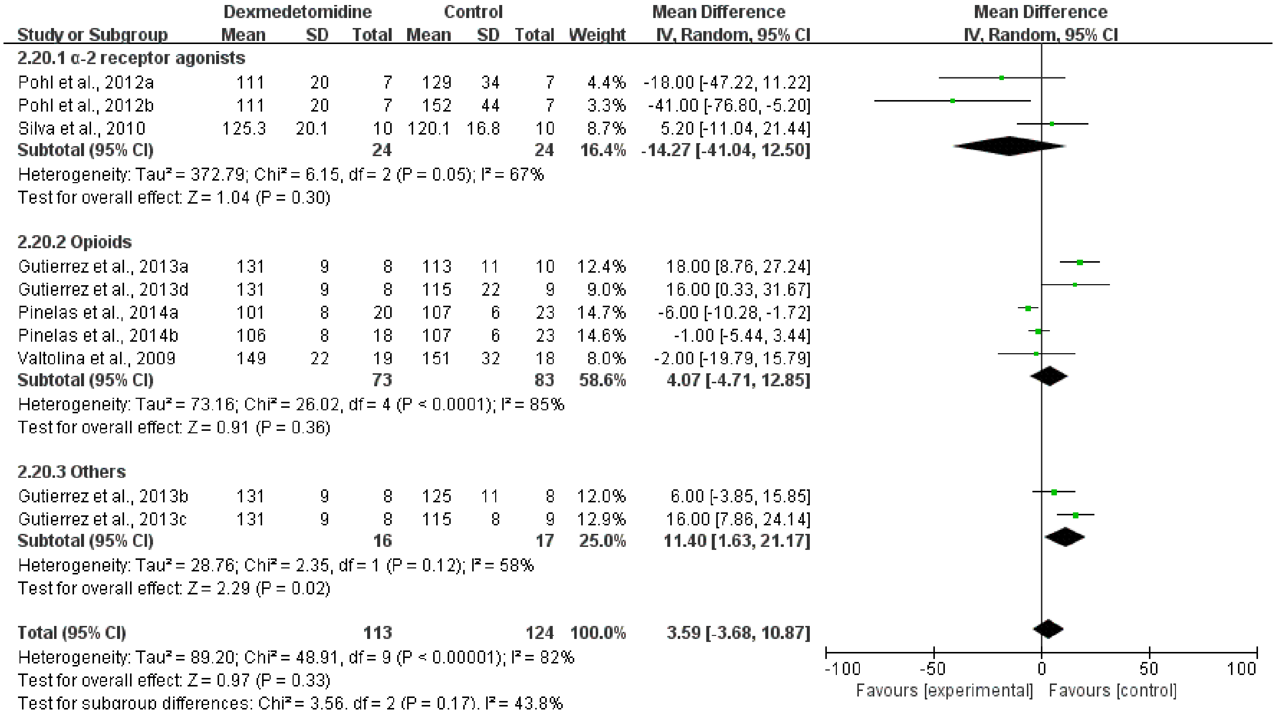

Figure S2 Forest plot of SAP at 60 min after premedication between dexmedetomidine and control group in balanced anesthesia.

Study or Subgroup	Dexmedetomidine Mean	SD	Total	Control Mean	SD	Total	Weight	Mean Difference IV, Random, 95% CI
**2.21.1 α-2 receptor agonists**								
Pohl et al., 2012a	91	18	7	100	18	7	8.1%	-9.00 [-27.86, 9.86]
Pohl et al., 2012b	91	18	7	119	39	7	3.8%	-28.00 [-59.82, 3.82]
Silva et al., 2010	106.5	15.8	10	99.9	14.9	10	11.7%	6.60 [-6.86, 20.06]
**Subtotal (95% CI)**			**24**			**24**	**23.5%**	**-5.95 [-23.59, 11.69]**
Heterogeneity: Tau² = 135.87; Chi² = 4.66, df = 2 (P = 0.10); I² = 57%								
Test for overall effect: Z = 0.66 (P = 0.51)								
**2.21.2 Opioids**								
Gutierrez et al., 2013a	112	5	8	95	11	10	16.9%	17.00 [9.35, 24.65]
Gutierrez et al., 2013d	112	5	8	90	17	9	13.2%	22.00 [10.37, 33.63]
Valtolina et al., 2009	103	14	19	99	20	18	13.6%	4.00 [-7.18, 15.18]
**Subtotal (95% CI)**			**35**			**37**	**43.7%**	**14.49 [4.98, 24.01]**
Heterogeneity: Tau² = 44.24; Chi² = 5.37, df = 2 (P = 0.07); I² = 63%								
Test for overall effect: Z = 2.98 (P = 0.003)								
**2.21.3 Others**								
Gutierrez et al., 2013b	112	5	8	107	10	8	16.8%	5.00 [-2.75, 12.75]
Gutierrez et al., 2013c	112	5	8	100	12	9	16.0%	12.00 [3.43, 20.57]
**Subtotal (95% CI)**			**16**			**17**	**32.8%**	**8.25 [1.41, 15.09]**
Heterogeneity: Tau² = 7.13; Chi² = 1.41, df = 1 (P = 0.24); I² = 29%								
Test for overall effect: Z = 2.36 (P = 0.02)								
**Total (95% CI)**			**75**			**78**	**100.0%**	**8.06 [1.25, 14.87]**
Heterogeneity: Tau² = 56.27; Chi² = 19.84, df = 7 (P = 0.006); I² = 65%								
Test for overall effect: Z = 2.32 (P = 0.02)								
Test for subgroup differences: Chi² = 4.08, df = 2 (P = 0.13), I² = 51.0%								

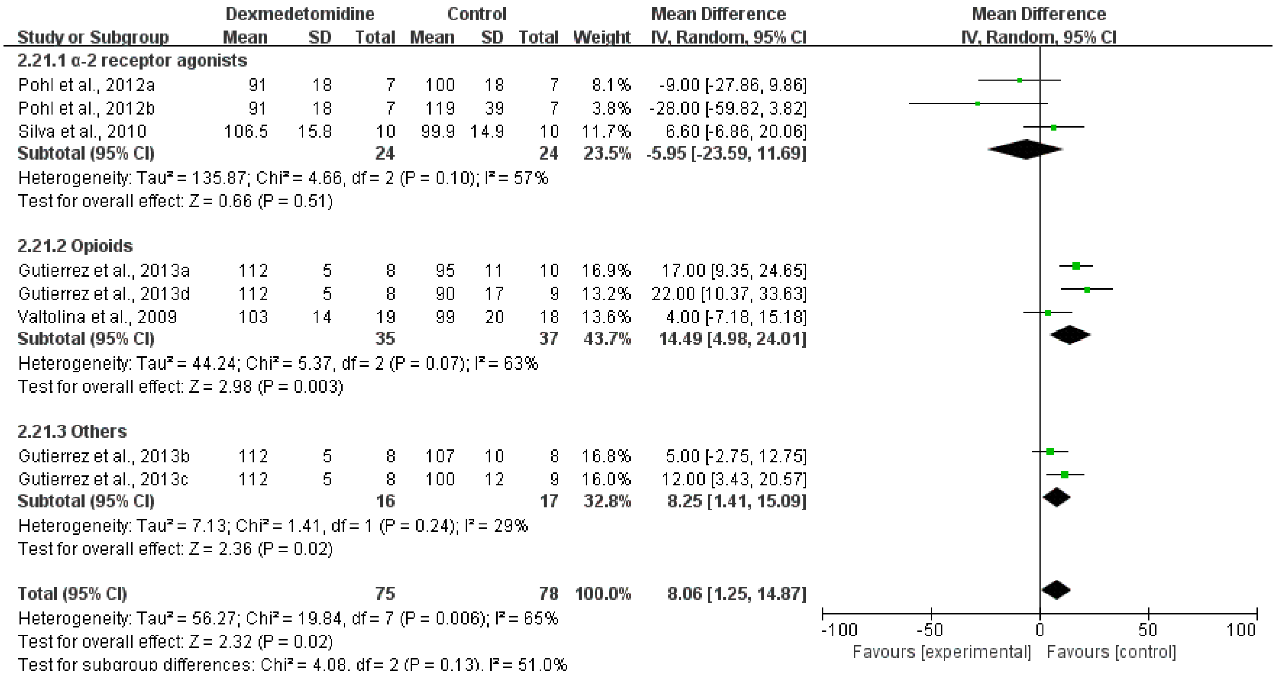

Figure S3. Forest plot of MAP at 60 min after premedication between dexmedetomidine and control group in balanced anesthesia.

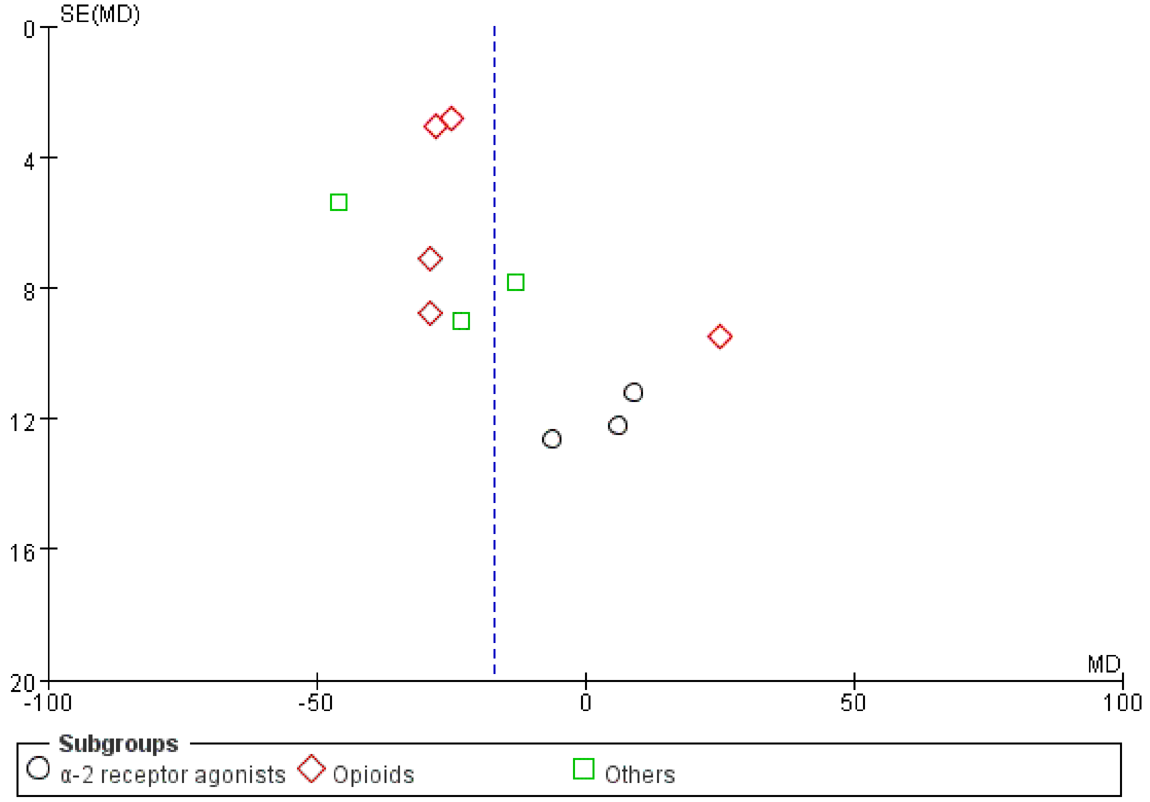

Figure S4. Funnel plot of HR at 60 min after premedication between dexmedetomidine and control group in balanced anesthesia.

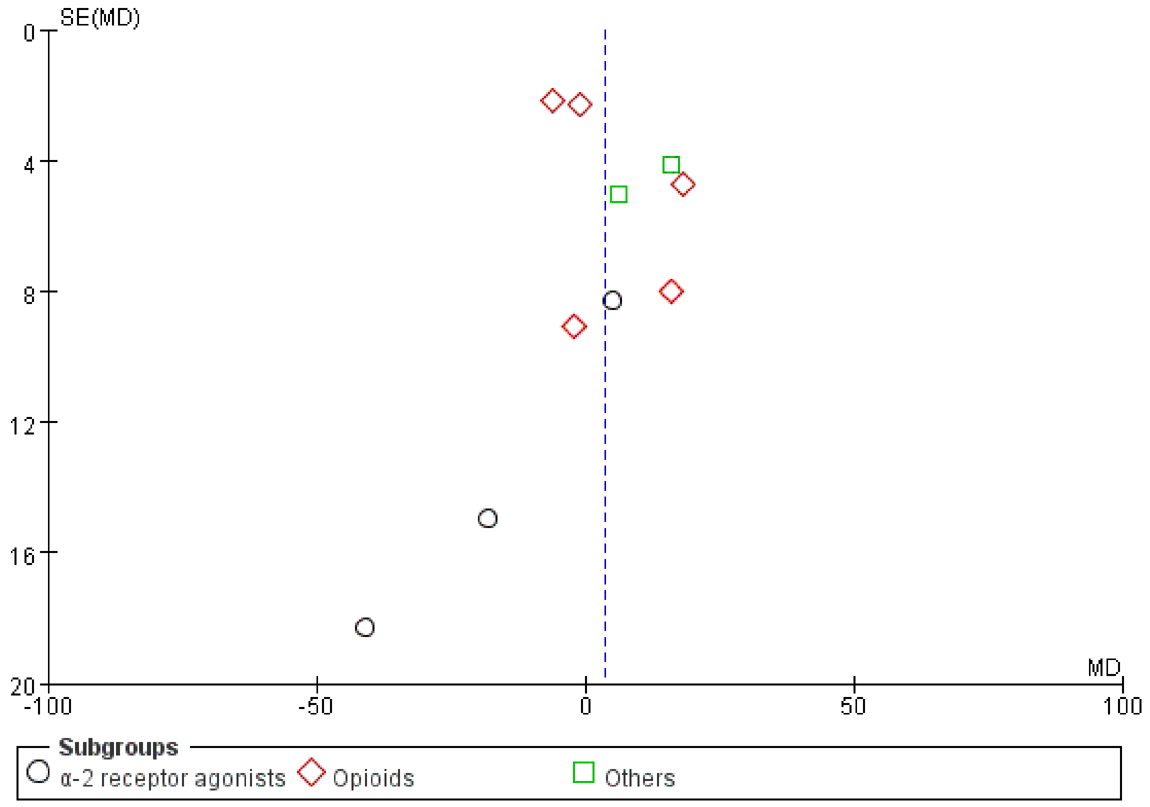

Figure S5. Funnel plot of SAP at 60 min after premedication between dexmedetomidine and control group in balanced anesthesia.

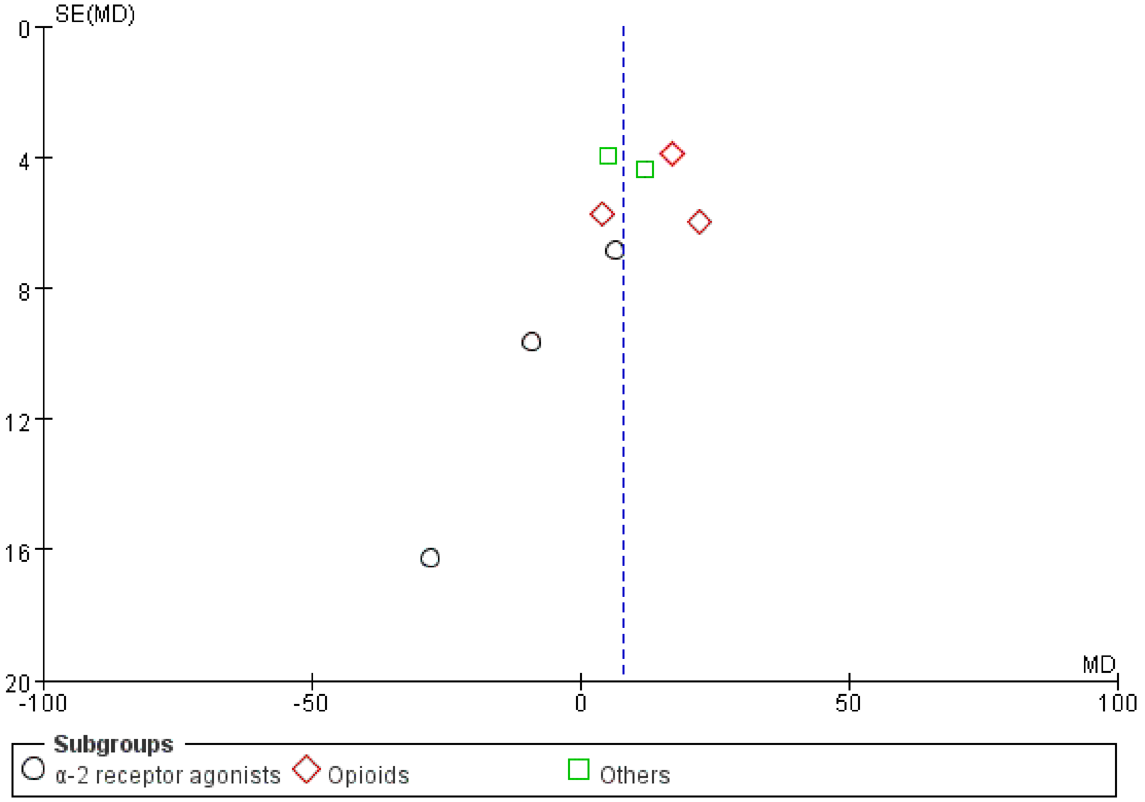

Figure S6. Funnel plot of MAP at 60 min after premedication between dexmedetomidine and control group in balanced anesthesia.
